# Maternal obesity and stillbirth at term; placental pathology—A case control study

**DOI:** 10.1371/journal.pone.0250983

**Published:** 2021-04-30

**Authors:** Hanna Åmark, Magnus Westgren, Meeli Sirotkina, Ingela Hulthén Varli, Martina Persson, Nikos Papadogiannakis

**Affiliations:** 1 Dept of Clinical Science and Education, Unit of Obstetrics and Gynecology, Karolinska Institute, Södersjukhuset, Stockholm, Sweden; 2 Dept of Clinical Sciences, Intervention & Technology, Karolinska Institute, Stockholm, Sweden; 3 Dep of Laboratory Medicine, Karolinska University Hospital Huddinge, Huddinge, Sweden; 4 Department of Women´s and Children´s Health, Karolinska Institutet, Stockholm, Sweden; 5 Dep of Medicine, Clinical Epidemiology Unit, Karolinska University Hospital, Stockholm, Sweden; 6 Dept of Clinical Science and Education, Unit of Pediatrics, Karolinska Institute, Södersjukhuset, Stockholm, Sweden; Univesity of Iowa, UNITED STATES

## Abstract

**Objective:**

The aim was to explore the potential role of the placenta for the risk of stillbirth at term in pregnancies of obese women.

**Methods:**

This was a case-control study comparing placental findings from term stillbirths with placental findings from live born infants. Cases were singleton term stillbirths to normal weight or obese women, identified in the Stockholm stillbirth database, n = 264 and n = 87, respectively. Controls were term singletons born alive to normal weight or obese women, delivered between 2002–2005 and between 2018–2019. Placentas were compared between women with stillborn and live-born infants, using logistic regression analyses.

**Results:**

A long and hyper coiled cord, cord thrombosis and velamentous cord insertion were stronger risk factors for stillbirth in obese women compared to normal weight women. When these variables were adjusted for in the logistic regression analysis, also adjusted for potential confounders, the odds ratio for stillbirth in obese women decreased from 1.89 (CI 1.24–2.89) to 1.63 (CI 1.04–2.56).

**Conclusion:**

Approximately one fourth of the effect of obesity on the risk of stillbirth in term pregnancies is explained by umbilical cord associated pathology.

## Introduction

Stillbirth is the main contributor to perinatal death in high income countries. The incidence of neonatal mortality has decreased while the incidence of stillbirth has remained stable over the last decades [1–5]. However, differences in prevalence of stillbirth between countries, and within countries [5, 6], indicate that it may be possible to further decrease the incidence of stillbirth. Maternal body mass index (BMI kg/m^2^) is an independent, modifiable risk factor for stillbirth, with an almost linearly increased risk of stillbirth with increasing BMI [1, 7, 8]. Obesity in pregnancy is increasingly prevalent world-wide [9]. The mechanisms behind the increased risk of stillbirth in obese women are largely unknown. It has been hypothesized that the changes in maternal metabolism [10] and the pro-inflammatory state associated with obesity may be contributing factors [11]. There is a positive association between increasing maternal BMI in early pregnancy and levels of C-peptide, a product from insulin synthetizing, and glucose level in cord blood at birth [12]. Both human and experimental studies support that fetal hyperglycemia and hyperinsulinemia are independent risk factors for fetal hypoxia [13]. Furthermore, the incidence of cord complications and chorioamnionitis is increased in obese women [14, 15], which may jeopardize fetal health. However, research on potential causes of the increased risk of stillbirth in obese women is scarce.

Pathological analysis of the placenta is important in the search for underlying causes of stillbirth [16, 17]. Lesions in the placenta and umbilical cord associated with stillbirth include signs of inflammation, signs of maternal circulatory disorders and umbilical cord complications [16, 18, 19]. However, it is unclear if these placental and umbilical cord findings are more common in stillbirths of obese pregnancies or if there are other placental findings characteristic of stillbirths in this group.

The aim of this study was to explore the potential mediating role of placental lesions on the causal pathway from maternal obesity to term stillbirth.

## Material and methods

This is a case control study. All singleton stillbirths diagnosed at term to normal weight women (BMI 18.5–24.9 kg/m2) or obese women (BMI ≥ 30 kg/m2), identified in the Stockholm Stillbirth database between the years 2002 and 2018 were selected as cases. The Stockholm stillbirth database contains detailed information on 1,694 stillbirths recorded between 2002 and 2018 [20, 21]. The Stockholm stillbirth database is part of the Swedish Pregnancy Register (Graviditetsregistret.se). Only fetuses without major malformations were included in this study. Pregnancies complicated by pre-gestational diabetes or gestational diabetes (GDM) were excluded in an attempt to investigate the effect of obesity on risk of stillbirth without concomitant diabetes. The information on each case of stillbirth registered in the database was prospectively collected and based on data from antenatal records, blood samples, chromosomal analyses, placental analyses, and fetal autopsy. Primary and secondary causes of death as well as the degree of certainty of the cause of death were established in a structured, consensus driven process [21]. All placental analyses were performed according to a systematized examination protocol comprising a two-step macroscopic and histologic evaluation and including standardized sampling, reporting and definition of histopathologic lesions, essentially in agreement with Amsterdam consensus criteria [22]. The protocol was established in 2000 and has been extensively described and implemented by our group [23–25]. For the purpose of the study, all placental analyses were re-evaluated by two senior perinatal pathologists.

Placentas from women with live born infants, the controls, were collected during two time periods. Between the years 2002 and 2005 a total of 200 term controls were collected. The first delivery after the delivery of a stillborn infant at the same delivery ward was chosen as a control. To ensure a large enough number of controls, another 219 controls were collected between October 2018 and December 2019. They were collected consecutively from 106 term, singleton pregnancies to women with obesity, and from 113 term pregnancies to normal weight women, delivered at Södersjukhuset, Stockholm, Sweden. Controls were collected both among women with planned cesarean section and at the delivery ward. To secure that the group of women with obesity was large enough, the controls collected between 2018 and 2019 were based on BMI.

Placentas from controls were sent fresh to the Section for Perinatal Pathology at Karolinska University Hospital, Huddinge. Placentas were analyzed by two senior perinatal pathologists following the same standardized protocol mentioned above. The pathologists were blinded for maternal BMI and other maternal characteristics. In case of uncertainty in the classification of one of the variables, the pathologists discussed the findings and came to a consensus conclusion.

Placentas were weighed trimmed, without membranes and umbilical cord, after fixation. Placental hyperplasia was defined as placental weight ≥ 90^th^ percentile for gestational age and placental hypoplasia was defined as placental weight ≤ 10^th^ percentile for gestational age, according to a standard curve [26]. Placental shape was evaluated as normal or abnormal, including presence of accessory lobes. Velamentous cord insertion was defined as cord insertion in the membranes, marginal cord insertion was defined as cord insertion located less than one centimeter from the placental edge; otherwise cord insertion was defined as central/paracentral. The umbilical cord was assessed as long, appropriate for gestational age or short/missing according to a standard curve [27]. Additional cord characteristics (coiling, presence of true knots, single umbilical artery) were recorded. Coiling index was estimated by dividing the number of vascular twists to the length of the cord; hyper coiling was defined as coiling index >0.3 and hypo coiling was defined as <0.07 [28]. Infarction of the placenta was defined histologically as ischemic necrosis of villi, its spread in the placenta was noted macroscopically. Villous size, configuration, architecture and size of the villous blood vessels as well as the trophoblast layer were assessed to evaluate degree of villous maturation. Villous maturation was defined as delayed when more than 50% of the villi examined in microscope were less mature than normal for gestational age. Chorioamnionitis was defined as infiltration of leukocytes extending into the amnion/chorion. It was assessed in a two-grade scale: stage 1, involvement of subchoreal fibrin layer or/and chorionic tissue; stage and stage 2 as involvement of amniotic membranes. Vasculitis was defined as presence of leukocytes in vessel walls in the chorionic plate or umbilical cord. Funisitis was defined as leukocyte infiltration in Wharton’s jelly. Vasculitis and funisitis are generally believed to represent signs of fetal inflammatory response [29]. Villitis was defined as mononuclear cell infiltration in villous stroma, its approximate spread in the placenta was assessed histologically. Thrombosis in the fetal circulation (stem villi, vessels in the chorionic plate and umbilical cord) was noted. The presence and degree of intervillous thrombosis was assessed histologically. The diagnosis of placental abruption was a combination of macroscopic and histologic signs and was based on the presence of retroplacental hemorrhage. Placental abruption with macroscopic and histologic signs are most commonly chronic since acute abruptio placenta often leave no signs. Additional histological features seen less often (chorangiosis, signs of hypoxia, increased erythropoiesis, villous edema, fibrin deposition, decidual arteriopathy) were also recorded.

Information on maternal characteristics were collected from the antenatal records. BMI was based on self-reported height and measured weight at the first antenatal visit during the first trimester of pregnancy. Maternal age at delivery was entered as a continuous variable in the statistical models. Parity was handled as a binominal variable, i.e. primipara yes/no. Smoking was recorded as yes/no at the first antenatal visit in the first trimester. Country of birth was handled as a binominal variable i.e. born in Sweden or not.

**Ethical approval** for this study was obtained from the Regional Research Ethics Committee at Karolinska institute in Stockholm, Sweden 2017/14-31/4 approved 9^th^ of March 2017, with amendment 2019–05991, approved 15^th^ of February 2020. According to the ethical approvement anonymized information about the cases came from the Stockholm Stillbirth database and mothers of the cases did not give an informed consent. All controls gave a written informed consent.

## Statistical analyses

Maternal characteristics were compared between cases of stillbirth and live born controls. Comparisons between continuous variables were done with Wilcoxon rank sum test, presented as median and interquartile range and comparisons between categorical variables were done with chi-square test, presented as proportions. Frequencies of the placental variables and birthweight were compared between cases and controls. Different causes of stillbirth were compared between pregnancies with and without obesity, using chi-square test. Since controls were selected with a pre-determined distribution of obesity, between 2018–2019, the proportion of obese women among these controls was corrected by weighting, to ascertain an equal proportion of obese women as in the general population. Data on the true proportions of normal weight women and obese women in the Stockholm region were collected from the National Board of Health and Welfare [30].

For each of the placental and umbilical cord variables we tested if the association between that variable and stillbirth differed between pregnancies of obese and normal weight women. Technically, we did this by fitting a linear regression model with the placental variable as the outcome, and stillbirth and obesity as covariates. We further included an interaction term between stillbirth and obesity and tested if it was statistically significant. We used robust standard errors to avoid assuming normality and homoscedasticity of the outcome [31]. Thereafter, a logistic regression analysis with obesity as exposure and stillbirth as outcome was conducted. Covariates in the logistic regression model were maternal age, smoking habits, parity and maternal country of birth. Preeclampsia and gestational hypertension were not added as potential mediators in the final model since they did virtually not affect the estimates. To be able to identify placental or umbilical cord characteristics potentially acting as mediators of the effect of obesity on the risk of stillbirth, different groups of placental variables, acting as potential mediators, were added to the adjusted logistic regression analysis, one at a time. These were; A) variables indicating an abnormal umbilical cord; velamentous or marginal umbilical insertion, an umbilical cord long for gestational age, umbilical cord thrombosis and a high coiling index, B) variables indicating inflammation i.e. chorioamnionitis stage 2, and C) variables indicating maternal circulatory disorders; retroplacental hemorrage, infarctions, increase amount of syncytial knots and decidual arteriopathy [16, 22]. We defined the proportion explained by a particular group of mediators as (log OR_1_-log OR_2_)/log OR_1_, where log OR_1_ is the log odds ratio for stillbirth from the model adjusted for potential confounders and log OR_2_ is the log odds ratio for stillbirth from the model additionally adjusted for one particular group of potential mediators. Bootstrap was used to calculate the confidence interval for the difference in log OR between analyses with and without potential placental/umbilical cord mediators. Bootstrap uses a sample distribution to approximate the population distribution so that the variance of the estimate can be obtained through a Monte-Carlo simulation by resampling from the sample [32]. Since there were missing values, especially for country of birth and parity, all analyses were re-run using multiple imputation to predict a value for all variables with missing values, using the default settings in the R package mice [33]. In addition, analyses were re-run with all possible two-way interactions.

## Result

The Stockholm stillbirth database contains information on 1,694 stillbirths delivered between 2002 and 2018. After exclusion of fetuses with major malformations, multiples, stillbirths diagnosed before gestational week 37+0 and fetuses to mothers with diabetes, our final cohort included 531 cases. 91 of these were born to obese women and 270 to women of normal weight. There were four cases of stillbirth to obese women with missing placenta and another six cases of stillbirth to normal weight women with missing placenta. In total there were n = 87 cases of stillbirths complicated by maternal obesity and n = 264 stillbirths to women of normal weight.

Maternal characteristics for live born and stillborn infants are shown in Table 1. A long umbilical cord was more common in obese women compared to normal weight women. The frequencies of different placental lesions compared between stillborn and live-born infants are shown in Table 2. A long and hyper coiled umbilical cord, vasculitis, velamentous or marginal cord insertion and umbilical cord thrombosis had a stronger association with stillbirth in obese women than in normal weight women. In pregnancies with stillbirth, the mean placental weight and birthweight were lower than in pregnancies with live births, independent of maternal obesity. Signs of more advanced acute and chronic inflammation, i.e. chorioamnionitis stage 2 and villitis grade 2, were associated with stillbirth in both pregnancies of normal weight and obese women, in addition villitis grade 3 was associated with stillbirth among normal weight women. Causes of term stillbirth according to the Stockholm stillbirth classification [21] did not differ between pregnancies of normal weight women and obese women (Table 3).

**Table 1:** Maternal characteristics, term live born and stillborn infants to normal weight and obese women.

Maternal characteristics	Normal weight live born n = 286	Normal weight stillborn n = 264	p-value	Obese live born n = 133	Obese stillborn n = 87	p-value
Age, years	31.79 (28,35)	32 (29,36)	0.16	32.63 (30.43,36)	33.5 (30,37)	0.872
BMI, kg/m2	21.98 (20.4,23.38)	22.34 (21.07,23.66)	0.014	32.41 (31.05,34.37)	31.56 (30.46,33.83)	0.114
Nulliparous (n, %)	157 (55.09%)	101 (43.53%)	0.013	55 (41.35%)	29 (38.67%)	0.817
Smoking (n,%)	13 (4.55%)	19 (7.25%)	0.327	1 (0.75%)	8 (9.2%)	0.006
Born in Sweden (n, %)	214 (82%)	156 (73.58%)	0.02	89 (70.63%)	43 (58.9%)	0.158
Gestational age, days	280 (274.75,285)	276 (267,283)	<0.001	281 (274.75,286.25)	280 (268,286)	0.309
Cesarean section, (n,%)	68 (25.66%)	22 (10.19%)	<0.001	61 (46.21%)	7 (9.86%)	<0.001
Gestational hypertension (n, %)	4 (1.4%)	2 (0.91%)	0.928	8 (6.02%)	2 (2.74%)	0.479
Pre-eclampsia (n, %)	3 (1.05%)	2 (0.91%)	1	2 (1.5%)	2 (2.74%)	0.931

Maternal characteristics for normal weight women (BMI 18.5–24.9 kg/m^2^) and obese women (BMI ≥30 kg/m^2^) with term live born and stillborn fetuses, respectively. For continuous variables medians with interquartile range are expressed and for categorical variables numbers and proportions are expressed.

**Table 2:** Placental and umbilical cord variables and birthweight; live born and stillborn infants to normal weight and obese women.

Placental and umbilical cord lesions	Normal weight live born n = 286	Normal weight stillborn n = 264	p-value	Obese live born n = 133	Obese stillborn n = 87	p-value
Fetal weight, g	3490 (3200,3817.5)	3200 (2880,3545)	<0.001	3615 (3290,3935)	3400 (2910,3717.5)	<0.001
SGA <10th percentile (n, %)	24 (8.39%)	63 (24.14%)	<0.001	8 (6.02%)	22 (25.29%)	<0.001
LGA >90th percentile (n, %)	33 (11.54%)	10 (3.83%)	0.003	21 (15.67%)	9 (10.34%)	0.486
Placental weight, g	480.5 (413.5,545.25)	423 (365,491)	<0.001	510.5 (449.5,580)	437 (376,522)	<0.001
Fetal weight/Placental weight	7.36 (6.8,8.15)	7.57 (6.68,8.57)	0.158	7.16 (6.52,7.75)	7.67 (6.53,8.75)	0.017
Placental hypoplasia (n, %)	37 (12.94%)	142 (53.79%)	<0.001	23 (17.16%)	41 (47.13%)	<0.001
Placental hyperplasia (n, %)	10 (3.5%)	15 (5.68%)	0.237	19 (14.29%)	3 (3.45%)	0.017
Abnormal shape of placenta (n, %)	39 (13.73%)	43 (16.6%)	0.416	13 (9.85%)	13 (15.66%)	0.29
Cord long for gw (n, %)	27 (9.44%)	81 (30.68%)	<0.001	26 (19.55%)	33 (37.93%)	0.004
Margin or velamentous cord insertion (n, %)	32 (11.27%)	35 (13.36%)	0.54	9 (6.82%)	19 (22.89%)	0.001
High coiling index (n, %)	5 (1.76%)	19 (7.39%)	0.003	3 (2.27%)	11 (13.41%)	0.003
Low coiling Index (n, %)	44 (15.49%)	39 (15.18%)	1	29 (21.97%)	11 (13.41%)	0.167
Umbilical cord both long and hyper coiled (n, %)	0 (0%)	8 (3.03%)	0.009	0 (0%)	8 (9.2%)	0.001
Umbilical cord knot (n, %)	0 (0%)	22 (8.46%)	0.003	4 (3.85%)	4 (4.88%)	1
Delayed villous maturation (n, %)	47 (16.49%)	101 (38.7%)	<0.001	31 (23.48%)	30 (35.71%)	0.073
Placental Infarction (n, %)	72 (23.68%)	69 (26.14%)	0.564	35 (26.12%)	21 (24.14%)	0.863
Thrombosis Placenta (n, %)	46 (16.14%)	65 (25%)	0.014	35 (26.52%)	30 (35.71%)	0.199
Thrombosis Umbilical cord (n, %)	31 (10.92%)	27 (10.38%)	0.939	12 (9.09%)	16 (19.05%)	0.055
Chorioamnionitis stage 1(n, %)	62 (21.75%)	52 (20%)	0.674	27 (20.45%)	18 (21.43%)	1
Chorioamnionitis stage 2(n, %)	33 (11.58%)	54 (20.77%)	0.005	10 (7.58%)	23 (27.38%)	<0.001
Vaskulitis Chorion plate (n, %)	34 (11.97%)	32 (12.21%)	0.954	11 (8.33%)	18 (21.84%)	0.008
Vaskulitis Cord (n, %)	15 (5.28%)	16 (6.06%)	0.834	7 (5.3%)	8 (9.2%)	0.399
Funisitis (n, %)	7 (2.46%)	15 (5.75%)	0.083	4 (3.05%)	8 (9.52%)	0.087
Villitis <1% (n, %)	19 (6.67%)	21 (8.08%)	0.65	11 (8.4%)	6 (7.23%)	0.961
Villitis 1–5% (n, %)	1 (0.35%)	9 (3.46%)	0.017	0 (0%)	6 (7.23%)	0.007
Villitis 5–30% (n, %)	0 (0%)	6 (2.31%)	0.03	0 (0%)	3 (3.61%)	0.111
Retroplacental hemorrage (n, %)	14 (4.91%)	27 (10.38%)	0.024	5 (3.79%)	5 (5.95%)	0.685

Placental characteristics, placental lesions and umbilical cord abnormalities for normal weight women (BMI 18.5–24.9 kg/m^2^) and obese women (BMI ≥30 kg/m^2^) with term live born and stillborn fetuses, respectively. For continuous variables medians with interquartile range are expressed and for categorical variables numbers and proportions are expressed.

**Table 3:** Primary cause of term stillbirth; lean and obese women.

Primary cause of stillbirth	Normal weight stillborn n = 264	Obese stillborn n = 87	P-value
Secure or probable cause of death (n, %)	123 (48.62%)	52 (61.9%)	0.047
Infection (n, %)	51 (20.08%)	20 (25%)	0.434
Feto-maternal transfusion (n, %)	9 (3.54%)	0 (0%)	0.19
Birth asphyxia (n, %)	4 (1.57%)	0 (0%)	0.589
Placental insufficiency/IUGR (n, %)	98 (38.58%)	27 (33.75%)	0.518
Umbilical cord complications (n, %)	33 (12.99%)	8 (10%)	0.606
Placental Abruptio (n, %)	20 (7.87%)	7 (8.75%)	0.988
Preeclampsia (n, %)	2 (0.79%)	3 (3.75%)	0.169
Intrahepatic cholestasis of pregnancy (n, %)	4 (1.57%)	0 (0%)	0.589
Coagulation disorder (n, %)	1 (0.39%)	0 (0%)	1
Other causes related to stillbirth (n, %)	6 (2.36%)	1 (1.25%)	0.874
Cause of stillbirth un-known (n, %)	25 (9.84%)	11 (13.75%)	0.438

Primary cause of stillbirth among normal weight women (BMI 18.5–24.9 kg/m^2^) and obese women (BMI ≥30 kg/m^2^). Birth asphyxia is defined as severe birth trauma or fetal monitoring suggesting fetal asphyxia, an intrapartal fetal death.

When the crude logistic regression was adjusted for the potential confounders maternal age, parity, country of birth and smoking, there was a reduction of the effect of obesity on the risk of stillbirth (Table 4). Placental variables, potentially mediating the effect of obesity on the risk of stillbirth were added to the adjusted logistic regression analysis, one group at a time, i.e. first umbilical cord abnormalities, then chorioamnionitis and then maternal circulatory disorders. When umbilical cord abnormalities were added to the logistic regression, the effect of obesity on the risk of stillbirth decreased with 24% (CI 0.04–0.92), from OR 1.89 (CI 1.24–2.89) to OR 1.63 (CI 1.04–2.56). Chorioamnionitis stage 2 or signs of maternal circulatory disorders did not significantly modify the effect of obesity on the risk of term stillbirth (Table 4). In total, for the logistic regression analyses adjusted for confounders, there were 18.5% of observations with missing values. Data on maternal country of birth was missing in 13% of the women, parity was missing in 5% of women, maternal age in 0.6% of women and information on smoking habits was missing in 1% of women. Thus, all analyses were re-run with multiple imputation of all missing data with virtually the same results, not shown. There were no significant interactions between placental variables on the association between maternal obesity and risk of stillbirth.

**Table 4:** Logistic regression models for the risk of stillbirth associated with obesity.

Logistic Regression obesity on Stillbirth	OR Obesity	95% CI	p-value	proportion of decrease of OR	95% CI for the proportion of decrease
Crude analyse obesity	2.18	1.5–3.19	<0.001		
Adjusted for age, parity, country of birth and smoking	1.89	1.24–2.89	0.003		
Confounders above and mediators; long cord, high coiling, umbilical cord thrombosis, velamentous/marginal insertion	1.63	1.04–2.56	0.03	-24%	(0.02–0.77)
Confounders above and mediator; chorioamnionitis stage 2	1.84	1.2–2.84	0.005	-4%	(-0.06–0.21)
Confounders above and mediators; retroplacental hemorrage, placental hypoplasia, infarcts, decidual arteriopathy, syncytial knots	1.78	1.11–2.86	0.02	-9%	(-0.271–0.67)

Results from the logistic regression model with stillbirth as outcome and obesity as exposure and in addition potential confounders added to the model and different groups of potential mediators, placental or umbilical cord covariates.

## Discussion

In the present study, umbilical cord abnormalities could explain approximately one fourth of the effect of obesity on the risk of stillbirth at term. However, the primary causes of stillbirth, according to the Stockholm stillbirth classification [21], did not differ between pregnancies with and without maternal obesity (Table 3).

The strengths of this study include the large number of consecutive cases of stillbirth, all evaluated according to the same protocol since 2002 [21]. Virtually all cases of stillbirth in the Stockholm region are included in the Stockholm Stillbirth database. Furthermore, placentas from all cases and all controls were evaluated by experienced perinatal pathologists, with few cases with missing placental analysis. Controls were collected for the purpose of this study, and not because of suspicion of pathology.

As stillbirth is a rare outcome, the absolute numbers included in this study are small although all cases of stillbirth over a period of 16 years were collected. Another limitation is the long time period during which cases were collected and the fact that controls were collected during two different time periods. During the two past decades the obstetric population has changed, and the obstetric care has improved which could introduce differences between cases and controls. The prevalence of obesity in pregnancy has increased during this time period [9]. However, the incidence of stillbirth has been stable over the last 20 years in Sweden, including in Stockholm and in obese women [3]. Hence, taking only exposure and outcome into account, year of birth is most likely not a confounder, also supported by the finding of unchanged statistical estimates when year of birth was accounted for in the analysis. However, for example the diagnostic criteria for gestational diabetes have changed during the time period of case collection [34]. As the criteria for GDM diagnosis today include lower glucose cut-off values than before, it is possible that some women without GDM a few years ago would have received a diagnosis of GDM today. The decision to exclude women with GDM or pregestational diabetes could potentially have selected obese women with a phenotype less sensitive to insulin resistance. This could lead to a selection of more healthy obese women which would potentially lead to smaller differences compared to the normal weight women. Stillborn infants had a lower birthweight than live born infants. This difference may be overestimated due to postmortem changes and the fact that the exact time-point for fetal death is not known. However, gestational age used in this calculation was the age at time of diagnosis, not the gestational age at which the baby was delivered. Additionally, this study only includes term stillbirths who probably are identified and diagnosed closer to the actual time of death than preterm stillbirths.

Umbilical cord complications are common causes of stillbirth [35]. In the current study, we found that both umbilical hyper coiling, velamentous or marginal cord insertion, umbilical cord thrombosis and a long cord were significant risk factors for stillbirth, in particular in pregnancies with maternal obesity. This is in line with results from previous studies [18, 19, 36]. A long cord could lead to impaired umbilical cord blood flow and an increased risk of fetal entanglement [19] and is also associated with increased risk of both umbilical thrombosis and umbilical knot. Why maternal obesity is associated with a longer umbilical cord is unclear. Data from animal studies suggest that cord length is at least partly determined by tensile forces, and fetuses with less amniotic fluid usually have shorter umbilical cords [37]. An increased risk of polyhydramnios in obese women [38], enabling the fetus´ to move more easily could be one explanation for the longer cords observed in obese pregnancies [38]. There is a positive association between maternal BMI and fetal weight [39], indicating a larger potential of growth for fetuses to obese women, which could potentially affect the growth of the umbilical cord too. The risk of umbilical cord thrombosis increases with a hyper coiled cord; both of which may impair cord blood flow and increase the risk of stillbirth [18]. Cord insertion is established in a very early phase of embryonal development [40], and it is thus likely that abnormal cord insertions are due to adverse impact in the very beginning of pregnancy in obese women.

A study analyzing cause-specific stillbirth from gestational week 16, stratified by maternal BMI, identified placental dysfunction, umbilical cord complications and hypertensive disorders as important risk factors for stillbirth in obese women [14]. However, pathophysiological mechanisms behind the increased risk of stillbirth in obese pregnancies are likely complex and may vary with length of gestation [14]. In the present study, there were no differences in primary cause of death in term stillbirths of women with and without obesity. However, it must be borne in mind that cause of stillbirth, especially at term, is often multifactorial and accurate determination of primary cause of death is not always straightforward. Moreover, the classification [21] is based on clinical criteria and experience and the specific significance of each contributing risk factor cannot always be exactly assessed or quantified. To what extent umbilical cord abnormalities contribute to the cause of stillbirth may differ between cases.

To what degree cord abnormalities may be detected in utero is unclear. Ultrasound is a good method for detection of abnormal cord insertion, although a larger proportion of abnormal cord insertions were correctly identified during the second trimester compared to the third trimester [41, 42]. Magnetic resonance imaging (MRI) has also been tested to measure umbilical cord length. However, measurements using MRI were unreliable with risk of overestimation of the umbilical cord length especially in case of long umbilical cords [43]. Research on determination of degree of coiling in utero is scarce, however ultrasound may be a possible method [28]. There is a need for development of techniques to more accurately identify umbilical cord abnormalities in utero. The stillbirth incidence is low and trying to predict stillbirth will lead to low positive predictive values. All possible predictive models will lead to investigations or extra surveillance of a large number of women among whom very few will suffer from stillbirth. Hence, methods of extra surveillance must be safe and not cause too much anxiety.

A previous study has showed that chorioamnionitis accounts for approximately 10% of the effect of obesity on the risk of stillbirth [15]. Our study showed that inflammation tended to be a mediator on the pathway from BMI to stillbirth. Obesity is associated with increased levels of inflammatory markers [44]. This in turn contributes to the increased risk of hypertensive disorders and other common complications in pregnancies with obesity [44]. It is also possible that inflammation associated with obesity contributes to the increased risk of stillbirth. In an animal model, it was demonstrated that high-fat-diet during pregnancy increased placental inflammation and decreased uteroplacental perfusion, regardless of obesity. High-fat-diet combined with obesity further decreased blood flow on the fetal side of the placenta and compromised placental function [45]. An association between chronic chorioamnionitis and preterm stillbirth potentially associated to maternal anti-fetal rejection has been found [46]. To what degree this could contribute also to term stillbirth is an open question. We hypothesized that a part of the increased risk of stillbirth in obese women could be explained by an increased risk of chronic fetal hypoxia. Maternal obesity is associated with fetal hyperinsulinemia, which is an independent risk factor for fetal hypoxia [12, 13]. However, the odds ratio for stillbirth in obese women remained unchanged when placental lesions associated with chronic hypoxia were adjusted for. This may be interpreted as chronic hypoxia not being an important risk factor for stillbirth in obese pregnancies or that ordinary placental analyses are too blunt to disclose more subtle changes of chronic hypoxia.

Continued research on mechanisms behind the increased risk of stillbirth among obese women is needed, as well as more knowledge about technics to identify abnormal cords in utero.

## Conclusion

Umbilical cord abnormalities may account for approximately one fourth of the effect of obesity on the risk of stillbirth at term.
